# Genetic Profiling and Phenotype Spectrum in a Chinese Cohort of Pediatric Cardiomyopathy Patients

**DOI:** 10.3390/jcdd12120466

**Published:** 2025-11-29

**Authors:** Guofeng Xing, Li Chen, Lizhi Lv, Guanyi Xu, Yabing Duan, Jiachen Li, Xiaoyan Li, Qiang Wang

**Affiliations:** 1 Department of Pediatric Cardiac Surgery, Beijing Anzhen Hospital, Capital Medical University, No. 2 Anzhen Street, Chaoyang District, Beijing 100029, China or xier2049@outlook.com (G.X.); or prolvlizhi@126.com (L.L.); helios_214029@163.com (Y.D.); lijiachen0915@126.com (J.L.); 2 Department of Pediatric Cardiology, Beijing Anzhen Hospital, Capital Medical University, No. 2 Anzhen Street, Chaoyang District, Beijing 100029, China; liliforever@163.com (L.C.); ligongligonglg@sina.com.cn (G.X.); 3 Beijing Institution of Heart Lung and Blood Vassal Diseases, Beijing Anzhen Hospital, Capital Medical University, No. 2 Anzhen Street, Chaoyang District, Beijing 100029, China

**Keywords:** pediatric, cardiomyopathies, WSE, genotype–phenotype correlation

## Abstract

This study examines pediatric cardiomyopathies by analyzing genetic and clinical data from 55 patients (2021–2024) at Beijing Anzhen Hospital. Four subtypes were studied: dilated (DCM, 24), hypertrophic (HCM, 22), arrhythmogenic right ventricular (ARVC, 7), and restrictive (RCM, 2). Clinical data, imaging, labs, and family histories were collected, with whole-exome sequencing (WES) identifying disease-causing variants classified via ACMG guidelines. Statistical analysis revealed a median age of 11 years, a proportion of 58% male participants, and ethnic diversity (21 northern Han, 29 southern Han, 5 minorities). In the cohort, 13 cases had an LVEF below 35%. Pathogenic/likely pathogenic (P/LP) variants were found in 21.8% of the patients, and variants of uncertain significance (VUS) were present in 38.2%, with *MYH7* (seven cases) and *MYBPC3* (five) being the most common. The WES positivity rates varied, at 58.3% (DCM), 72.7% (HCM), and 33.3% (ARVC/RCM). DCM patients with P/LP/VUS variants showed better contractile function (Fractional Shortening: 29.0% vs. 16.5%, *p* = 0.008). Females in the DCM group had poorer cardiac function (lower LVEF, higher LVESd, lower cardiac output) compared to males, with more females (nine vs. three) exhibiting an LVEF < 35% (*p* = 0.041). No significant gender differences were observed in the HCM cases. These findings highlight genotype–phenotype correlations and underscore the need for early intervention in female DCM patients.

## 1. Introduction

Cardiomyopathies, which encompass a spectrum of disorders including DCM, HCM, ARVC, RCM, and non-dilated left ventricular cardiomyopathy (NDLVC), are characterized by structural and functional heart muscle abnormalities [1].

To elaborate, DCM is characterized by the dilatation of LV and global or regional systolic dysfunction unexplained solely by abnormal loading conditions or CAD. HCM, on the other hand, is defined as the presence of increased LV wall thickness or mass that is not solely explained by abnormal loading conditions [1]. ARVC primarily involves dilatation of the RV and/or dysfunction in the presence of histological involvement and/or electrocardiographic abnormalities. Lastly, RCM, the rarest pediatric cardiomyopathy, is defined as restrictive left and/or RV pathophysiology in the presence of normal or reduced diastolic volumes, regular or reduced systolic volumes, and typical ventricular wall thickness [1,2].

In the last 20 years, significant progress has been made in the identification of disease-causing genes and the phenotype–genotype spectrum in cardiomyopathies. Nowadays, more than 37 genes have been identified as being associated with cardiomyopathies, and genetic tests have been recommended as a standard of care for clinical management according to the latest guidelines [3]. Due to complex etiological and pathogenic factors, different variants or even the same mutation in one gene can end up in different types of cardiomyopathies. For the DCM group, the etiology is highly heterogeneous; variants in *TTN* (OMIM*188840), *MYH7* (OMIM*160760), *RBM20* (OMIM*613171), and *DSP* (OMIM*125647) are associated [4]. In HCM cases, about half of them are inherited as a Mendelian genetic trait and they primarily exhibit autosomal dominant inheritance [5]. A proportion of 60% of pediatric HCM cases are related to sarcomere protein disease [6], in which the most common variants are in *MYBPC3* (OMIM*600958), *MYH7*, *TNNT2* (OMIM*191045), *TNNI3* (OMIM*191044), *FLNC* (OMIM*102565), *PLN* (OMIM*172405), *MYL2* (OMIM*160781), and *MYL3* (OMIM*160790) [7].

On the other hand, research on ARVC and RCM is relatively rare. Variants in *PKP2* (OMIM*602861), *DSC2* (OMIM*125645), and *DSG2* (OMIM*125671) play an important role in ARVC [8]. And RCM is more closely associated with *TNNI3*, *TNNT2*, *DES* (OMIM*125660), and *ACTC1* (OMIM*102540) [9]. 

It is worth noting that pathogenic genes are not consistent between adult patients and pediatric patients, which is due to natural selection, suggesting that the pathogenic factors contributing to cardiomyopathies in children are more complex and diverse. As one of the most severe pediatric cardiovascular diseases, cardiomyopathies persist as highly complex disorders with a high incidence rate (1 per 100,000 children) [10], but due to difficulties relating to studying children, most research has focused on adult patients [11,12]. Research on genotype–phenotype analysis of cardiomyopathies in children remains scarce.

In this study, a genotype–phenotype analysis of 55 pediatric cardiomyopathy patients who underwent WES was performed to explore the clinical features and prognosis of pediatric cardiomyopathy.

## 2. Materials and Methods

The cohort comprised 68 case records of patients diagnosed with cardiomyopathy at the Pediatric Heart Center of Beijing Anzhen Hospital between January 2021 and September 2024 who completed WES with the informed consent of their guardians. A total of 13 cases with uncertain diagnoses or inherited arrhythmias were excluded to avoid the interference. This cohort of 55 pediatric cardiomyopathy patients comprised 24 patients with DCM, 22 with HCM, 7 with ARVC, and 2 with RCM. Clinical diagnoses were made based on the latest guidelines for the management of cardiomyopathies issued by the European Society of Cardiology (ESC) [1].

In this study, echocardiography, cardiac magnetic resonance imaging (MRI), and laboratory test results for CK, CK-MB, hsTnI, CREA, BNP, and NT-proBNP were collected for each patient from their medical or hospitalization records, available at our center. Furthermore, follow-up interviews were conducted via telephone to gather information on patients’ prognoses and family genetic histories.

Genomic DNA was extracted from the patient’s peripheral blood samples. The IDT kit 1.0 was used for whole-exome capture, and sequencing was performed on a Hiseq 4000 (Illumina, San Diego, CA, USA). Variants—detected using the GATK v4.6.1.0 software—were interpreted according to the American College of Medical Genetics and Genomics.

The paired-end reads were aligned to the reference human genome build hg19 using bwa-mem. Raw variants were called using GATK, and functional and database annotation was performed with SnpEff. The ClinVar database (release 20180225) was used to clinically interpret variants. Sequences with less than 10× coverage and SNVs with <35% heterozygous ratio and base call quality score ≤ Q20 were not considered for the analysis.

Continuous variables are expressed as the mean values, and categorical variables are depicted using frequency. For different groups, continuous variables were compared using the *t*-test, and categorical variables were compared using the χ^2^ test or Fisher’s exact test. All statistical analyses in this study were performed using IBM SPSS Statistics 29.0.1.0. A two-sided *p* value < 0.05 indicated statistical significance.

This study followed the STROBE (Strengthening the Reporting of Observational Studies in Epidemiology) guidelines for observational research, and the completed checklist is available in the Supplementary Materials.

## 3. Results

### 3.1. Baseline Characteristics and Clinical Features

**Figure 1:**
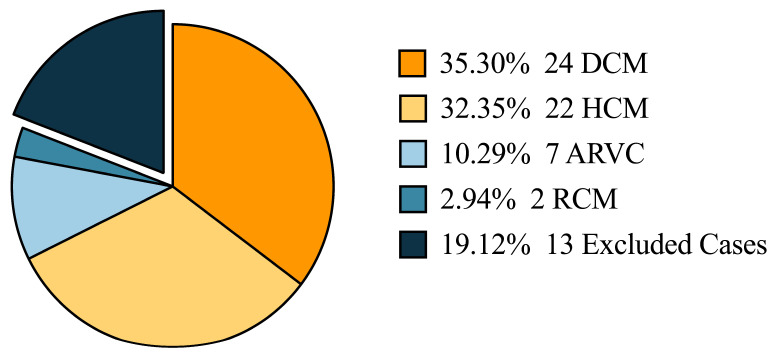
The proportion of different diseases in the cohort.

The final analysis included 55 cases in the cohort, including 24 patients with DCM, 22 with HCM, 7 with ARVC, and 2 with RCM (Figure 1).

The median age of the patients is 11 years old, with the youngest being 3 months old and the oldest 36 years old. The proportion of male patients varied from 44.4% to 77.3% in different groups. In total, 21 patients belonged to the Han ethnic group from northern regions, 29 to the Han ethnic group from southern areas, and 5 to ethnic minorities such as the Mongolians; 41 of the 55 patients (74.5%) originated from underdeveloped regions. In total, 32 of the 55 patients were successfully followed up with, resulting in a loss to follow-up rate of 41.8%. Among the 55 patients, 6 had a family history of cardiomyopathy. A patient was implanted with an implantable cardioverter-defibrillator (ICD), and another patient was a heart transplant recipient (Table 1).

The average left ventricular ejection fractions (LVEFs) in the DCM, HCM, and ARVE/RCM groups are 41.3%, 70.1%, and 60.0%, and thirteen cases have an LVEF lower than 35%. Three cases have congenital heart defects, including atrial septal defect (ASD) or patent foramen ovale (PFO), nine patients have non-sustained ventricular tachycardia (NSVT), and two cases have AV blocks.

### 3.2. Genetic Testing Outcomes

**Figure 2:**
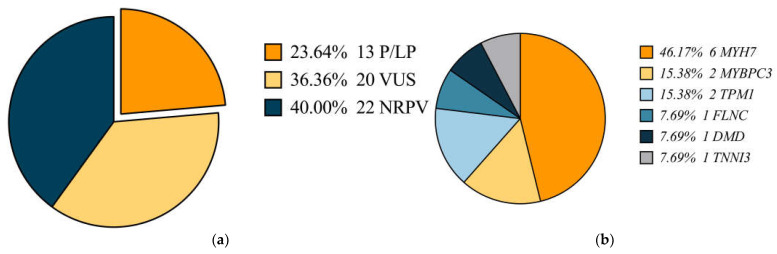
(**a**) The proportion of different ACMG classifications of cases in the cohort. (**b**) The proportion of different genotypes in the P/LP cases. (*MYH7* = Myosin Heavy Chain 7, *MYBPC3* = Myosin Binding Protein C3, *TPM1* = Tropomyosin 1, *FLNC* = Filamin C, *DMD* = Dystrophin, and *TNNI3* = Troponin I Type 3).

In total, 13 patients have Pathogenic or Likely Pathogenic (P/LP) variants, 21 patients have Variant of Uncertain Significance (VUS) variants, and 22 have no relevant pathogenic gene variants (WES-Negative). Five patients simultaneously carry two genetic variants, with one carrying both LP and VUS variants (Figure 2a).

In the DCM group, two cases have been identified with P/LP variants in the *MYH7* (c.3667G>A) and *DMD* genes (*DMD* deletion exon 48–51).

The nine cases in the HCM group identified with P/LP variants include four cases in *MYH7* (*MYH7* c.2770G>A, *MYH7* c.2207T>C, *MYH7* c.1193G>A, *MYH7* c.2791_2793del), two cases in *MYBPC3* (*MYBPC3* c.1504C>T, *MYBPC3* c.2065C>T), one case in *FL*NC (OMIM*102565) (*FLNC* c.6031G>A), and two cases in *TPM1* (*TPM1* c.574G>A, *TPM1* c.64G>A). The 11 VUS variants in the HCM group comprised 3 in the *MYBPC3* gene, 2 in the *FLNC* gene, 2 in the *MYH7* gene, and 4 in other genes.

There are also two cases in the ARVC/RCM group that were identified with P/LP variants, including one in *MYH7* (*MYH7* c.1357C>G) and another in *TNNI3* (c.574C>T), as well as one VUS in the *SCN5A* gene.

**Figure 3:**
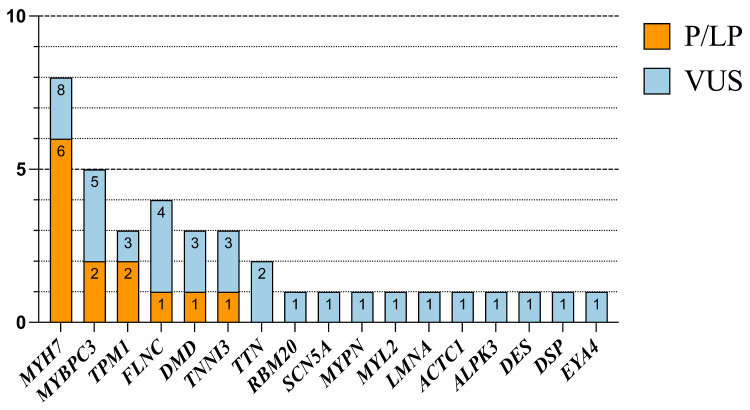
The number of P/LP and VUS cases of different ACMG classifications in different genotypes.

Variants in *MYH7* were detected in all three groups, and variants in *MYBPC3* and *FLNC* were detected in both the DCM and HCM groups. The details of the variants in each case of all three groups are shown in Figure 2b and Figure 3, and Table A1, Table A2 and Table A3.

### 3.3. Comparing the Differences Between P/LP and VUS and WES-Negative Cases

The average age of the cohort is 11.0 years, with the average being 11.8 years for the P/LP and VUS cases, and 10.6 years for the WES-Negative patients (*p* = 0.462). The ratio of male patients is 22/25 (88.0%) in the P/LP and VUS group and 10/20 (50.0%) in the WES- Negative group (*p* = 0.165). Thirteen cases have an LVEF lower than 35%, with nine in the P/LP and VUS group and four in the WES-Negative group (*p* = 0.199) (Table 2).

In the DCM group, the median age is 11.5 years for P/LP and VUS cases and 11.0 years for WES-Negative patients (*p* = 0.541). Only one patient in the P/LP and VUS group reported a family history of cardiomyopathies. Twelve cases have an LVEF lower than 35%, with eight in the P/LP and VUS group and four in the WES-Negative group. The average FS (Fractional Shortening) in the P/LP and VUS group is 28.2%, and 18.1% in the WES-Negative patients (*p* = 0.008) (Table A4).

For the HCM group, the median age is 10.5 years for the P/LP and VUS cases and 11.5 years for the WES-Negative patients (*p* = 0.455). Five cases have a reported family history of cardiomyopathies, all of which are in the P/LP and VUS group. The average LVESd in the P/LP and VUS group is 20.6 mm and it is 19.8 mm in the WES-Negative patients (*p* = 0.033) (Table A5).

There are no statistically significant differences in the other aspects, such as the LVEF, CO, LVEDd, ESV, and maximum left ventricular wall thickness among the subgroups in both the DCM and HCM groups (Table A5).

### 3.4. Comparing Gender Differences in DCM Group

The differences in disease severity between Male and Female cases in each group have been compared, which are especially notable in the DCM group. In this group, there are 11 male cases and 13 female cases; the average age is 10.5 years for male patients and 9.3 years for female patients (*p* = 0.511). In the male patient group, there are four cases of NYHA/ROSS I, three cases of NYHA/ROSS II, four cases of NYHA/ROSS III, and no cases of NYHA/ROSS IV. In the female patient group, there are no cases of NYHA/ROSS I, seven cases of NYHA/ROSS II, two cases of NYHA/ROSS III, and four cases of NYHA/ROSS IV. The average LVEF in the male group is 49.4% and 35.1% in the female group (*p* = 0.055). Twelve cases have an LVEF lower than 35%, with three in the male group and nine in the female group (*p* = 0.041). The average CO is 4.2 L/min in the male group and 2.8 L/min in the female group (*p* = 0.038); the average LVESd is 32.9mm in the male group and 44.1mm in the female group (*p* = 0.023). There are no statistically significant differences in the other aspects, such as the LVEDd, EDV, and ESV (Table 3).

## 4. Discussion

Genetic testing is gaining prominence in cardiomyopathy diagnostics, driven by guideline integration and technological advancements. Increasing numbers of cases now undergo genetic evaluation, underscoring its critical role in disease understanding, diagnosis, and management, and emphasizing the need for comprehensive testing in cardiomyopathy patients [13].

Investigating genotype–phenotype associations in pediatric cardiomyopathy, particularly dilated (DCM) and hypertrophic (HCM) forms, poses complex challenges due to low disease incidence and multifactorial genetic/environmental influences, complicating pediatric diagnosis [11,12,14].

For pediatric patients, genomic testing remains optional, with parental decisions influenced by financial constraints and awareness levels. Notably, WES testing in China is currently not covered by medical insurance, making it a self-funded procedure. The high cost often leads families to forgo this diagnostic tool, potentially introducing bias in gene prevalence research due to underrepresentation of economically disadvantaged groups. This structural limitation highlights the need for equitable access policies in genomic medicine. Our study circumvented this by focusing solely on mutation-positive patients, ensuring unbiased comparisons of severity.

In this study, the genotype distribution aligns broadly with that in prior reports, though the overall genetic testing positivity rate (45.5%) and subtype-specific rates were significantly lower than international benchmarks (60%) [7,8,15,16]. This discrepancy likely stems from variations in the clinical application of testing criteria.

Gender disparities in cardiomyopathy severity and prognosis were observed in the DCM group [17,18,19,20]. The female patients in this cohort exhibited more severe clinical features, including a higher proportion with an LVEF < 35%, lower CO, and larger LVESd at diagnosis (all statistically significant). The males showed a trend toward a higher LVEF, though this was not statistically significant, likely due to limited sample size. These findings may reflect gender-based differences in cardiomyopathy diagnosis, with females experiencing more pronounced disease severity upon symptom onset. Due to small cohort sizes and admission bias, undiagnosed females may exhibit milder severity; therefore, longitudinal follow-up is crucial to explore gender differences in disease trajectory, considering social factors and delayed care. Consequently, early intervention for at-risk females remains clinically valuable.

Focusing on various disease groups, in the DCM group, the analysis only indicates statistical differences in the LVEF and FS, with P/LP and VUS cases showing better systolic function (*p* = 0.060 for LVEF, *p* = 0.008 for FS). This indicates that among the DCM patients, those carrying P/LP and VUS genetic variants may have exhibited better left ventricular function, which is consistent with reports from other studies [21,22]. This suggests that different pathogenic genes may play relatively more significant roles in DCM pathogenesis to varying degrees, highlighting potential differences in disease severity and underscoring the need for further research. It also emphasizes the critical importance of precise genotype identification for stratified management of DCM patients.

The HCM group has the highest positive rate of P/LP variant detection compared to the DCM and ARVC/RCM groups. However, the analysis revealed no statistically significant differences in key clinical parameters between the P/LP VUS and WES-Negative subgroups.

No significant genotype–phenotype associations were detected in either group, likely due to cardiomyopathy’s low incidence, limited genetic testing uptake in patients, and constrained data availability. Additionally, unclear criteria for genetic testing indications may contribute to this. Thus, adhering to guidelines for genetic testing and counseling in suspected inherited cardiovascular diseases is critical during diagnosis and treatment [4]. Patients with variants of uncertain significance (VUS) require regular reassessment and updated reporting.

Notably, the cohort included a unique DCM case with a DES gene variant of uncertain significance (VUS) (c.335T>A). Desmin, the key intermediate filament protein in muscle cells, links organelles and desmosomes. Pathogenic DES variants cause skeletal and cardiac myopathies, but most missense variants lack functional validation. Studies have shown that N-terminal 1A domain variants disrupt filament assembly, leading to cytoplasmic Desmin aggregation, while C-terminal variants maintain a normal filamentous structure [23]. As in the case of this patient—who developed rapid cardiac decline following fulminant myocarditis, failed conservative treatment, and underwent heart transplantation in June 2024 with currently stable recovery—this supports the association between DES c.335T>A variants and susceptibility to acute fulminant myocarditis. These findings underscore the need for functional validation of DES variants to clarify their pathogenic potential and clinical implications [24].

## 5. Conclusions

In conclusion, exploring genotype–phenotype correlations in pediatric cardiomyopathy, particularly DCM and HCM, remains a multifaceted challenge due to the rarity of these conditions and their complex interplay with genetic and environmental factors. Genetic testing, increasingly integrated into diagnostic guidelines, is pivotal but yields less favorable rates in our cohort than international benchmarks, highlighting the need for better understanding and application of test indications. Gender differences in DCM disease severity underscore the importance of early intervention in female patients. While genotype–phenotype analysis in pediatric patients is limited by data scarcity, our findings suggest potential differences in disease severity based on genetic variants. Notably, a unique case involving a DCM patient with a DES gene VUS and fulminant myocarditis underscores the role of genetic variants in disease susceptibility and progression. Further research is warranted to elucidate these complex correlations and optimize patient management strategies.

## Figures and Tables

**Table 1:** Baseline characteristics of total population, according to phenotype.

	Total Population (*N* = 55)	DCM (*N* = 24)	HCM (*N* = 22)	ARVC/RCM (*N* = 9)
Age, y	11.3 ± 6.7	9.9 ± 4.7	13.2 ± 8.9	11.0 ± 4.2
Male	32	11	17	4
Family history	6	1	5	0
NYHA/ROSS I	14	4	8	2
NYHA/ROSS II	26	10	12	4
NYHA/ROSS III	11	6	2	3
NYHA/ROSS IV	4	4	0	0
LVEF ≤ 35%	13	12	1	1
LVEF, %	54.2 ± 20.6	41.3 ± 18.0	70.1 ± 10.3	60.0 ± 19.2
LVEDd, mm	46.5	51.0	40.0	47.1
NSVT	9	3	0	6
AV-blocks	2	1	0	1
RASS-I	5	1	2	2
Beta-blockers	6	2	4	0
Diuretics	7	2	3	2
Digoxin	7	3	2	2
ICD implantation	1	0	0	1
RFCA	4	0	0	4
Surgery	5	2	3	0
Heart Transplantation	1	1	0	0

Values are average or n. ICD = implantable cardioverter-defibrillator; LVEF = left ventricular ejection fraction; NSVT = non-sustained ventricular tachycardia; NYHA = New York Heart Association; ROSS = Ross Heart Failure Classification; RAAS-I = renin–angiotensin–aldosterone system inhibitors.

**Table 2:** Phenotype of all cases, according to ACMG classification.

	Total Population (*N* = 55)	P/LP and VUS (*N* = 25)	WES-Negative (*N* = 20)	*p* Value
Age	11.0	11.8	10.6	0.462
Male	32	22	10	0.165
NYHA/ROSS I	14	10	4	
NYHA/ROSS II	26	15	11	
NYHA/ROSS III	11	4	7	
NYHA/ROSS IV	4	4	0	
LVEF ≤ 35%	13	9	4	0.199

Values are average or n.

**Table 3:** The phenotype of DCM, according to gender.

	Total Population (*N* = 24)	Male (*N* = 11)	Female (*N* = 13)	*p* Value
Age	9.8 (11.0)	10.5 (11.0)	9.3 (11.0)	0.511
Chest congestion	7	2	5	0.276
I	4	4	0	
II	10	3	7	
III	6	4	2	
IV	4	0	4	
LVEF, %	41.3 (34.3)	49.4 (52.5)	35.1 (30.0)	0.055
LVEF ≤ 35%	12	3	9	0.041
FS, %	24.3 (24.0)	28.2 (28.5)	20.8 (18.0)	0.052
CO, L/min	3.4 (3.4)	4.2 (4.2)	2.8 (2.5)	0.038
LVEDd, mm	50.7 (51.0)	46.2 (44.5)	54.1 (56.0)	0.089
LVESd, mm	39.2 (39.0)	32.9 (32.0)	44.1 (45.0)	0.023
EDV, mL	145.9 (138.9)	143.6 (153.1)	147.9 (134.4)	0.866
ESV, mL	108.7 (109.8)	92.9 (88.1)	122.3 (124.2)	0.311

Values are average or n. EDV = End-Diastolic Volume; ESV = End-Systolic Volume; FS = Fractional Shortening; LVEDd = Left Ventricular End-Diastolic Diameter; LVESd = Left Ventricular End-Systolic Diameter.

## Data Availability

The datasets generated and/or analyzed during the current study are available in the China National Center for Bioinformation repository (www.cncb.ac.cn) under accession number PRJCA043437.

## Supplementary Materials

The following supporting information can be downloaded at: https://www.mdpi.com/article/10.3390/jcdd12120466/s1; STROBE Statement—Checklist.

## Abbreviations

The following abbreviations are used in this manuscript:

DCM	Dilated Cardiomyopathy
HCM	Hypertrophic Cardiomyopathy
ARVC	Arrhythmogenic Right Ventricular Cardiomyopathy
RCM	Restrictive Cardiomyopathy
NDLVC	Non-dilated Left Ventricular Cardiomyopathy
MRI	Magnetic Resonance Imaging
WES	Whole-Exome Sequencing
ACMG	American College of Medical Genetics and Genomics
ESC	European Society of Cardiology
P/LP	Pathogenic or Likely Pathogenic
VUS	Variant of Uncertain Significance
LVEF	Left Ventricular Ejection Fraction
FS	Fractional Shortening
CO	Cardiac Output
LVEDd	Left Ventricular End-Diastolic Diameter
LVESd	Left Ventricular End-Systolic Diameter
EDV	End-Diastolic Volume
ESV	End-Systolic Volume
IVS	Interventricular Septum
MTLVW	Maximum Thickness of the Left Ventricular Wall

## Appendix A

**Table A1:** Details of variants in DCM group.

Patient ID	Age at Diagnosis	Gene/ Transcript/Exon	Locus	Variants	Interpretation	ACMG Evidence
D01	13	*DES* NM_001927.3 exon1	chr2:220283519	c.335T>A p.L112Q	VUS	PM6_Supporting + PM2_Supporting + PP3
D04	16	*TTN* NM_001267550 exon250	chr2:179484500	c.46544del p.N15515fs	VUS	PVS1 + PM2_Supporting
D07	3	*TNNI3* NM_000363.4 exon6	chr19:55666189	c.292C>T p.R98X	VUS	PM2_Supporting + PP5
D08	11	*FLNC* NM_001458.4 exon18	chr7:128483506	c.2686G>A p.G896R	VUS	PP3 + BA1
D11	9	*ALPK3* NM_020778.4 exon7	chr15:85402543	c.4493G>A p.C1498Y	VUS	PM2_Supporting + PP2
D13	6	*DMD* NM_004006.3 exon48-51	chrX:31792077-31893490	deletion(exon48-51) 630bp	P	PVS1 + PS4 + PM2_Supporting
D14	14	*LMNA* NM_170707.3 exon2	chr1:156100539	c.488A>T p.H163L	VUS	PM1_Supporting + PM2_Moderate + PP2_Supporting
D17	16	*TTN* NM_001267550. 2 intron327	chr2:179423067	c.87118 + 1G>A splicing	VUS	PVS1 + PM2_Supporting + PP5_Supporting
D18	11	*TPM1* NM_001018005 exon3	chr15:63349235	c.292G>A p.E98K	VUS	PM1_Supporting + PM2_Moderate + PP3_Supporting
D19	4	*MYPN* NM_032578.3 exon15	chr10:69955256	c.3125G>A p.R1042H	VUS	PM2
		*DSP* NM_004415.3 exon17	chr6:7574921	c.2329A>G p.I777V	VUS	PM2 + BP6
D21	13	*DMD* NM_004006.2 exon42	chrX:32328383	c.5933G>A p.R1978H	VUS	BS1 + BS2
D22	13	*EYA4* NM_004100.4 exon12	chr6:133802712	c.1082C>G p.S361C	VUS	PM2
D23	0.3	*MYH7* NM_000257.3 exon27	chr14:23889113	c.3667G>A p.E1223K	LP	PM2_Moderate + PM1_Supporting + PP2 + PP3 + PP5
D24	10	*TNNI3* NM_000363.4 exon7	chr19:55665438	c.509G>A p.R170Q	VUS	PS2 + PS4_Moderate + PM2_Supporting + PP3

**Table A2:** Details of variants in HCM group.

Patient ID	Age at Diagnosis	Gene/ Transcript/Exon	Locus	Variants	Interpretation	ACMG Evidence
H01	15	*MYH7* NM_000257.3 exon38	chr14:23883310	c.5561C>T p.T1854M	VUS	PM2_Moderate + PP3_Supporting + PP2_Supporting
H02	9	*MYBPC3* NM_000256.3 exon7	chr11:47369442	c.787G>A p.G263R	VUS	BS2
H03	9	*TPM1* NM_001018005.1 exon6	chr15:63353922	c.574G>A p.E192K	LP	PS4_Strong + PM2_Supporting + PP1 + PP3_Moderate + PP2_Supporting
H05	13	*MYH7* NM_000257.3 exon23	chr14:23893268	c.2770G>A p.E924K	P	PS2_Strong + PS3_Supporting + PM1_Moderate + PM2_Moderate + PM5_Moerate + PP2 + PP3
H07	10	*MYBPC3* NM_000256.3 exon17	chr11:47364249	c.1504C>T p.R502W	P	PS4 + PP1_Strong + PS3_Moderate + PM5 + PM2_Supporting + PP3
H08	19	*MYBPC3* NM_000256.3 exon21	chr11:47361204	c.2065C>T p.Q689X	LP	PVS1 + PM2_Supporting
H09	10	*MYH7* NM_000257.3 exon20	chr14:23894983	c.2207T>C p.I736T	P	PS4 + PM1 + PP1_Moderate + PM2_Supporting + PP2 + PP3
H10	14	*MYH7* NM_000257.3 exon13	chr14:23898502	c.1193G>A p.G398E	LP	PM1 + PS4_Supporting + PM2_Supporting + PP2 + PP3
		*MYH7* NM_000257.3 exon23	chr14:23893145	c.2893G>T p.E965X	VUS	PVS1_Moderate + PM2_Moderate
H13	10	*ACTC1* NM_005159.4 exon6	chr15:35083350	c.955A>G p.I319V	VUS	PM2_Moderate + PM1_Supporting + PP2_Supportting
H14	8	*FLNC* NM_001458.4 exon37	chr7:128492908	c.6031G>A p.G2011R	LP	PM6_Strong + PS4_Supporting + PM2_Supporting + PP3
H15	17	*TPM1* NM_001018005.1 exon1	chr15:63335092	c.64G>A p.A22T	LP	PS4 + PM2 + PM1_Supporting + PP2_Supporting + PP3_Supporting
H16	11	*MYBPC3* NM_000256.3 exon23	chr11:47360106	c.2273G>A p.G758D	VUS	PS4 + PM2 + PP3_Supporting
		*MYL2* NM_000432.3 exon4	chr12:111352057	c.207G>A p.M69I	VUS	PM2 + PP2_Supporting + PP3_Supporting
H17	10	*FLNC* NM_001458.4 exon30	chr7:128489556	c.5123C>T p.A1708V	VUS	PM2 + PP3_Supporting
		*DMD* NM_004006.2 exon30	chrX:32429964	c.4138C>T p.H1380Y	VUS	PM2 + BP4_Supporting
H19	17	*MYBPC3* NM_000256.3 exon19	chr11:47362757	c.1829A>T p.D610V	VUS	PM2 + PP3 + PM5_Supporting
H21	0.6	*MYH7* NM_000257.4 exon23	chr14:23893245- 23893247	c.2791_2793del p.E931del	LP	PP1_Strong + PS4_Moderate + PM4_Supporting + PM2_Supporting
H22	12	*FLNC* NM_001458.4 exon14	chr7:128482387	c.2224A>G p.I742V	VUS	PM2
		*RBM20* NM_0011343 exon2	chr10:112541347	c.980G>A p.G327D	VUS	PM2 + BP6_Supporting

**Table A3:** Details of variants in ARVC and RCM group.

Patient ID	Age at Diagnosis	Gene/ Transcript/Exon	Locus	Variants	Interpretation	ACMG Evidence
A04	16	*SCN5A* NM_000335.4 exon24	chr3:38598727	c.4291A>T p.R1431W	VUS	PS4_Strong + PM2_Supporting + PP3 + PM1_Moderate + PM5_Supporting
R01	6	*TNNI3* NM_000363.4 exon8	chr19:55663261	c.574C>T p.R192C	LP	PS2 + PM5 + PM2_Supporting + PP3
R02	7	*MYH7* NM_000257.4 exon14	chr14:23898214	c.1357C>G p.R453G	LP	PM1 + PM2_Supporting + PM5 + PM6 + PP3

**Table A4:** Phenotypes of DCM, according to ACMG classification ^1^.

	Total Population (*N* = 24)	P/LP and VUS (*N* = 16)	WES-Negative (*N* = 8)	*p* Value
Age	9.8 (11.0)	10.3 (11.5)	9.0 (11.0)	0.541
Male	11	9	2	0.148
Family history	1	1	0	
Chest congestion	7	5	2	0.751
NYHA/ROSS I	4	4	0	
NYHA/ROSS II	10	5	5	
NYHA/ROSS III	6	3	3	
NYHA/ROSS IV	4	4	0	
LVEF, %	41.3 (34.3)	45.7 (51.0)	33.1 (30.0)	0.060
LVEF ≤ 35%	12	8	4	1.000
FS, %	24.3 (24.0)	28.2 (29.0)	18.1 (16.5)	0.008
CO, L/min	3.4 (3.4)	3.5 (3.6)	3.4 (3.3)	0.926
LVEDd, mm	50.7 (51.0)	48.6 (43.0)	54.5 (51.5)	0.229
LVESd, mm	39.2 (39.0)	36.3 (36.0)	44.6 (43.0)	0.116
EDV, mL	145.9 (138.9)	138.3 (138.9)	162.9 (162.5)	0.359
ESV, mL	108.7 (109.8)	94.9 (109.7)	139.8 (133.3)	0.141

^1^ Values are average or n. EDV = End-Diastolic Volume; ESV = End-Systolic Volume; FS = Fractional Shortening; LVEDd = Left Ventricular End-Diastolic Diameter; LVESd = Left Ventricular End-Systolic Diameter.

**Table A5:** Phenotypes of HCM, according to ACMG classification ^1^.

	Total Population (*N* = 24)	P/LP and VUS (*N* = 16)	WES-Negative (*N* = 8)	*p* Value
Age	13.2 (10.5)	13.9 (10.5)	11.2 (11.5)	0.455
Male	17	12	5	
Family history	5	5	0	
Chest congestion	5	3	2	
NYHA/ROSS				
I	8	5	3	
II	12	10	2	
III	2	1	1	
IV	0	0	0	
LVEF, %	70.1 (74.0)	69.9 (74.6)	70.5 (71.0)	0.926
FS, %	39.5 (39.0)	40.1 (37.0)	38.7 (41.0)	0.637
CO, L/min	5.1 (5.3)	5.1 (5.4)	5.2 (5.3)	0.913
LVEDd, mm	39.7 (40.0)	43.3 (43.0)	34.3 (32.5)	0.062
LVESd, mm	23.5 (25.0)	26.0 (26.0)	19.8 (18.5)	0.033
EDV, mL	116.6 (100.5)	122.4 (106.4)	103.1 (94.5)	0.552
ESV, mL	41.9 (30.4)	48.1 (48.0)	27.5 (29.1)	0.094
IVS, mm	16.5 (15.0)	13.6 (15.0)	21.7 (15.6)	0.251
MTLVW, mm	20.9 (15.0)	20.7 (16.0)	21.4 (23.3)	0.943

^1^ Values are average or n. EDV = End-Diastolic Volume; ESV = End-Systolic Volume; FS = Fractional Shortening; LVEDd = Left Ventricular End-Diastolic Diameter; LVESd = Left Ventricular End-Systolic Diameter. IVS = Interventricular Septum; MTLVW = Maximum Thickness of the Left Ventricular Wall.

## References

[1] Arbelo E., Protonotarios A., Gimeno J.R., Arbustini E., Barriales-Villa R., Basso C., Bezzina C.R., Biagini E., Blom N.A., De Boer R.A. (2023). 2023 ESC Guidelines for the management of cardiomyopathies. Eur. Heart J..

[2] Elliott P., Andersson B., Arbustini E., Bilinska Z., Cecchi F., Charron P., Dubourg O., Kühl U., Maisch B., McKenna W.J. (2008). Classification of the cardiomyopathies: A position statement from the European Society of Cardiology Working Group on Myocardial and Pericardial Diseases. Eur. Heart J..

[3] Wilde A.A.M., Semsarian C., Márquez M.F., Sepehri Shamloo A., Ackerman M.J., Ashley E.A., Sternick E.B., Barajas-Martinez H., Behr E.R., Bezzina C.R. (2022). European Heart Rhythm Association (EHRA)/Heart Rhythm Society (HRS)/Asia Pacific Heart Rhythm Society (APHRS)/Latin American Heart Rhythm Society (LAHRS) Expert Consensus Statement on the State of Genetic Testing for Cardiac Diseases. Heart Rhythm..

[4] Mazzarotto F., Tayal U., Buchan R.J., Midwinter W., Wilk A., Whiffin N., Govind R., Mazaika E., de Marvao A., Dawes T.J.W. (2020). Reevaluating the Genetic Contribution of Monogenic Dilated Cardiomyopathy. Circulation.

[5] Ommen S.R., Ho C.Y., Asif I.M., Balaji S., Burke M.A., Day S.M., Dearani J.A., Epps K.C., Evanovich L., Ferrari V.A. (2024). 2024 AHA/ACC/AMSSM/HRS/PACES/SCMR Guideline for the Management of Hypertrophic Cardiomyopathy: A Report of the American Heart Association/American College of Cardiology Joint Committee on Clinical Practice Guidelines. Circulation.

[6] Lipshultz S.E., Orav E.J., Wilkinson J.D., Towbin J.A., Messere J.E., Lowe A.M., Sleeper L.A., Cox G.F., Hsu D.T., Canter C.E. (2013). Risk stratification at diagnosis for children with hypertrophic cardiomyopathy: An analysis of data from the Pediatric Cardiomyopathy Registry. Lancet.

[7] Ingles J., Goldstein J., Thaxton C., Caleshu C., Corty E.W., Crowley S.B., Dougherty K., Harrison S.M., McGlaughon J., Milko L.V. (2019). Evaluating the Clinical Validity of Hypertrophic Cardiomyopathy Genes. Circ. Genom. Precis. Med..

[8] James C.A., Jongbloed J.D.H., Hershberger R.E., Morales A., Judge D.P., Syrris P., Pilichou K., Domingo A.M., Murray B., Cadrin-Tourigny J. (2021). International Evidence Based Reappraisal of Genes Associated with Arrhythmogenic Right Ventricular Cardiomyopathy Using the Clinical Genome Resource Framework. Circ. Genom. Precis. Med..

[9] Brodehl A., Gerull B. (2022). Genetic Insights into Primary Restrictive Cardiomyopathy. J. Clin. Med..

[10] Lipshultz S.E., Law Y.M., Asante-Korang A., Austin E.D., Dipchand A.I., Everitt M.D., Hsu D.T., Lin K.Y., Price J.F., Wilkinson J.D. (2019). Cardiomyopathy in Children: Classification and Diagnosis: A Scientific Statement From the American Heart Association. Circulation.

[11] Paldino A., Dal Ferro M., Stolfo D., Gandin I., Medo K., Graw S., Gigli M., Gagno G., Zaffalon D., Castrichini M. (2022). Prognostic Prediction of Genotype vs Phenotype in Genetic Cardiomyopathies. J. Am. Coll. Cardiol..

[12] Murray B., James C.A. (2022). Genotype-phenotype Correlates in Arrhythmogenic Cardiomyopathies. Curr. Cardiol. Rep..

[13] Butnariu L.I., Russu G., Luca A.-C., Sandu C., Trandafir L.M., Vasiliu I., Popa S., Ghiga G., Bălănescu L., Țarcă E. (2024). Identification of Genetic Variants Associated with Hereditary Thoracic Aortic Diseases (HTADs) Using Next Generation Sequencing (NGS) Technology and Genotype–Phenotype Correlations. Int. J. Mol. Sci..

[14] Curran L., de Marvao A., Inglese P., McGurk K.A., Schiratti P.-R., Clement A., Zheng S.L., Li S., Pua C.J., Shah M. (2023). Genotype-Phenotype Taxonomy of Hypertrophic Cardiomyopathy. Circ. Genom. Precis. Med..

[15] Richard P., Charron P., Carrier L., Ledeuil C., Cheav T., Pichereau C., Benaiche A., Isnard R., Dubourg O., Burban M. (2003). Hypertrophic cardiomyopathy: Distribution of disease genes, spectrum of mutations, and implications for a molecular diagnosis strategy. Circulation.

[16] Kaski J.P., Norrish G., Gimeno Blanes J.R., Charron P., Elliott P., Tavazzi L., Tendera M., Laroche C., Maggioni A.P., Baban A. (2024). Cardiomyopathies in children and adolescents: Aetiology, management, and outcomes in the European Society of Cardiology EURObservational Research Programme Cardiomyopathy and Myocarditis Registry. Eur. Heart J..

[17] Akinrinade O., Lesurf R., Lougheed J., Mondal T., Smythe J., Altamirano-Diaz L., Oechslin E., Mital S., Genomics England Research Consortium (2023). Age and Sex Differences in the Genetics of Cardiomyopathy. J. Cardiovasc. Transl. Res..

[18] Siontis K.C., Ommen S.R., Geske J.B. (2019). Sex, Survival, and Cardiomyopathy: Differences Between Men and Women with Hypertrophic Cardiomyopathy. J. Am. Heart Assoc..

[19] Cannatà A., Fabris E., Merlo M., Artico J., Gentile P., Pio Loco C., Ballaben A., Ramani F., Barbati G., Sinagra G. (2020). Sex Differences in the Long-term Prognosis of Dilated Cardiomyopathy. Can. J. Cardiol..

[20] Rowin E.J., Maron M.S., Wells S., Patel P.P., Koethe B.C., Maron B.J. (2019). Impact of Sex on Clinical Course and Survival in the Contemporary Treatment Era for Hypertrophic Cardiomyopathy. J. Am. Heart Assoc..

[21] Tayal U., Prasad S.K. (2017). Myocardial remodelling and recovery in dilated cardiomyopathy. JRSM Cardiovasc. Dis..

[22] Bertero E., Fracasso G., Eustachi V., Coviello D., Cecconi M., Giovinazzo S., Toma M., Merlo M., Sinagra G., Porto I. (2023). Diagnostic yield and predictive value on left ventricular remodelling of genetic testing in dilated cardiomyopathy. ESC Heart Fail..

[23] Brodehl A., Holler S., Gummert J., Milting H. (2022). The N-Terminal Part of the 1A Domain of Desmin Is a Hot Spot Region for Putative Pathogenic DES Mutations Affecting Filament Assembly. Cells.

[24] Ammirati E., Raimondi F., Piriou N., Sardo Infirri L., Mohiddin S.A., Mazzanti A., Shenoy C., Cavallari U.A., Imazio M., Aquaro G.D. (2022). Acute Myocarditis Associated with Desmosomal Gene Variants. JACC Heart Fail..

